# Sustainable Biomass Glucose-Derived Porous Carbon Spheres with High Nitrogen Doping: As a Promising Adsorbent for CO_2_/CH_4_/N_2_ Adsorptive Separation

**DOI:** 10.3390/nano10010174

**Published:** 2020-01-19

**Authors:** Yao Li, Shiying Wang, Binbin Wang, Yan Wang, Jianping Wei

**Affiliations:** 1 School of Safety Science and Engineering, Henan Polytechnic University, Jiaozuo 454000, Chinayanwang@hpu.edu.cn (Y.W.); 2 State Key Laboratory Cultivation Base for Gas Geology and Gas Control, Henan Polytechnic University, Jiaozuo 454000, China; 3 School of Materials Science and Engineering, Henan Polytechnic University, Jiaozuo 454000, China

**Keywords:** N-doped porous carbon spheres, sustainable biomass glucose, CO_2_ activation and urea treatment, gas adsorptive separation, IAST selectivity

## Abstract

Separation of CO_2_/CH_4_/N_2_ is significantly important from the view of environmental protection and energy utilization. In this work, we reported nitrogen (N)-doped porous carbon spheres prepared from sustainable biomass glucose via hydrothermal carbonization, CO_2_ activation, and urea treatment. The optimal carbon sample exhibited a high CO_2_ and CH_4_ capacity, as well as a low N_2_ uptake, under ambient conditions. The excellent selectivities toward CO_2_/N_2_, CO_2_/CH_4_, and CH_4_/N_2_ binary mixtures were predicted by ideal adsorbed solution theory (IAST) via correlating pure component adsorption isotherms with the Langmuir−Freundlich model. At 25 °C and 1 bar, the adsorption capacities for CO_2_ and CH_4_ were 3.03 and 1.3 mmol g^−1^, respectively, and the IAST predicated selectivities for CO_2_/N_2_ (15/85), CO_2_/CH_4_ (10/90), and CH_4_/N_2_ (30/70) reached 16.48, 7.49, and 3.76, respectively. These results should be attributed to the synergistic effect between suitable microporous structure and desirable N content. This report introduces a simple pathway to obtain N-doped porous carbon spheres to meet the flue gas and energy gas adsorptive separation requirements.

## 1. Introduction

With the rapid development of modern society, fossil fuels such as coal and petroleum are always maintaining a heavy demand. Inevitably, the burning of fossil fuels emits a large amount of the greenhouse gas carbon dioxide (CO_2_), which has led to a pressing environmental burden [1,2,3]. Approximately, 30% of CO_2_ emissions in the atmosphere comes from fossil fuel-based power plants [4]; thus, it is essential to capture and separate CO_2_ from flue gases (typically containing ~85% N_2_ and 15% CO_2_) to limit it exhausting into the atmosphere [5,6]. Methane (CH_4_) has a much higher global warming potential (GWP) than that of CO_2_, with a greenhouse effect 21 times that of CO_2_ and a power of damage for ozone (O_3_) 7 times that of CO_2_ [6,7]. In addition, CH_4_ is also a clean and high-caloric-energy gas, which is considered as alternative energy sources to replace petroleum and coal. Coalbed methane (CBM) is one kind of unconventional natural gas with a main composition of CH_4_, which has abundant reserves in China [8,9]. However, the utilization rate of CBM is only about 40% in China, because low-concentration CBM (CH_4_ less than 30%) is usually directly emitted into the atmosphere as drainage gas, which is not only a waste of energy sources but also pollutes the environment [9]. Therefore, it is urgent to concentrate on CH_4_ in CBM for effective utilization [10]. Apart from CH_4_, CBM also contains certain CO_2_ and N_2_, which would significantly cause pipeline and equipment corrosion and reduce the heating value of the CBM [8,11]. Thus, separation of CO_2_ and N_2_ from CH_4_ is highly demanded in order to effectively utilize the low-concentration CBM and alleviate atmospheric contamination.

Nowadays, many technologies have been applied for gas separation/purification, including membrane separation, cryogenic distillation, hydrate crystallization, chemical absorption, and physical adsorption-based methods. In the abovementioned technologies, pressure swing adsorption (PSA) as a promising technology has received intense interest due to its great advantages of high energy efficiency, low investment costs, and ease of control [5,6,9,12]. However, the property of adsorbents is the core of PSA technology, playing an important role in gas adsorption and separation. Up to now, a variety of porous adsorbents have been developed for the adsorptive separation of CO_2_/N_2_, CO_2_/CH_4_, and CH_4_/N_2_ binary mixtures, such as metal−organic frameworks (MOFs) [9,13], zeolites [3,14], porous organic polymers (POPs) [15], and carbon-based materials including carbon nanotubes [16,17], graphene [18], and activated porous carbon [19,20,21,22,23]. Among them, porous carbon absorbents have manifested many advantages, including easy preparation, low cost, large surface area, controllable porosity and surface functionality, hydrophobicity, and resistance to both bases and acids. One attractive aspect for porous carbon adsorbents is that they can be prepared by using various cheap carbon precursors, such as waste plastic polyethylene terephthalate [19], carbon black [24,25], coal [26,27,28], oil sands coke [29], and various biomass [30,31,32]. Among these precursors, biomass materials stand out for their environmental friendliness, wide availability, low cost, and renewability, and have been extensively used as a precursor for the preparation of gas-selective adsorbents. For example, our previous work reported one waste wool-derived porous carbon with equimolar binary mixtures CO_2_/CH_4_ and CH_4_/N_2_ with a selectivity of 3.19 and 7.62, respectively, at 25 °C and 1 bar [31]. Yang et al. prepared porous carbon by KOH activation from shrimp shells for efficient CO_2_ capture and CO_2_/N_2_, CO_2_/CH_4_, and CH_4_/N_2_ separation [32]. Fan et al. synthesized a cost-effective carbonaceous sorbent from coconut shells by KOH activation, which exhibited a high CO_2_ capacity of 4.26 mmol g^−1^ and CO_2_/N_2_ selectivity of 29 at 25 °C under atmospheric pressure [33]. Cao et al. manufactured three-dimensional porous carbon frameworks with an outstanding CO_2_ capture and CO_2_/N_2_ selectivity by the KOH-assisted hydrothermal method from mangosteen peel waste [34]. In other words, previous works have indicated that biomass-derived porous carbons are promising adsorbents for CO_2_/CH_4_/N_2_ adsorption and separation. Nevertheless, the preparation of biomass-derived porous carbons for gas-selective adsorption usually uses the harsh activator KOH, which is undesirable due to its strong inherent causticity, causing equipment corrosion and damage [35,36]. Thus, it is crucial to use a mild activation reagent of environmentally friendly nature for further development of gas-selective separation applications. Of various substances used as activators, CO_2_ as a physical activation agent is a good alternative, which is not toxic but can effectively etch carbon precursors to produce a microporous structure [36]. 

The adsorptive separation of CO_2_/N_2_, CO_2_/CH_4_, and CH_4_/N_2_ when using porous carbons as adsorbents is mainly based on both equilibrium and kinetic adsorption. Considering the gas mixture with very close physical properties (such as the same polarity and similar molecular diameter), the adsorbents should satisfy particular requirements, e.g., narrow pore size distribution (PSD) and well-polarized frameworks [37]. From a kinetic perspective, recent studies have reported that adsorbents with well-defined micropores have promising properties for separating small gas molecules that are similar in size [10,37,38]. On the other hand, the polarity of the adsorbent framework could enable weak interactions between the gas molecules and the polar channels, which may further help to separate the gas mixture [23,37]. Nitrogen (N)-doping is an effective method to increase the polarity of carbon frameworks [37,39], which has been reported a lot in CO_2_/CH_4_/N_2_ selective adsorption [21,23,37,40,41]. However, nowadays, most porous carbon is fine powder (<30 μm) belonging to Geldart’s group C classification [42,43]. When the carbon powders applicate in industrial dynamic systems, such as fixed/fluidized bed reactors, facing the challenge of plug formation, channeling, and agglomeration because of cohesive forces (such as van der Waals, electrostatic, and moisture-induced surface tension forces) existing between particles [44,45]. Inspired by Raganati and his co-workers, temperature-controlled [46] and sound-assisted [47,48,49] fluidization can be used to achieve a fluidization regime of these cohesive particles, which makes such fine carbon powders not only easily testable for characterization in static analysis systems, but also capable of actual use in dynamic systems.

**Figure 1:**
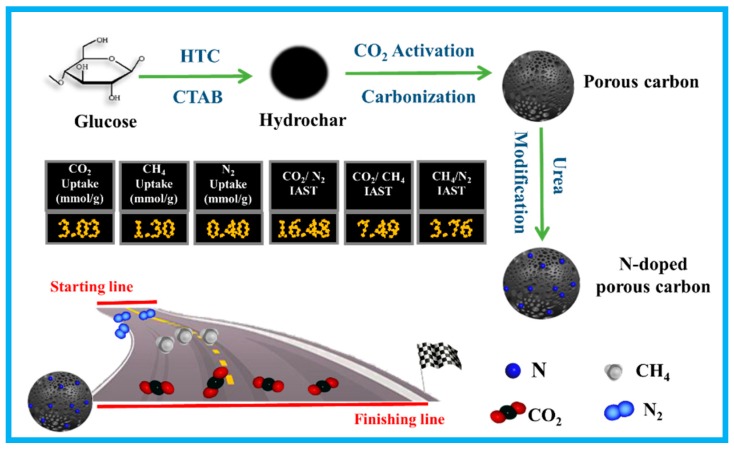
Schematic of the preparation of glucose-based carbon spheres and their applications.

Considering these pros and cons, in this work, we prepare N-doped porous carbon spheres for efficient CO_2_/CH_4_/N_2_ adsorptive separation by using sustainable biomass glucose as the raw material, environmentally friendly CO_2_ as the activator, and urea as the nitrogen agent. As illustrated in Figure 1, the carbon-rich precursor glucose is first converted into hydrochar spheres with the presence of cetyltrimethyl ammonium bromide (CTAB) by using the hydrothermal carbonization treatment. Then, one-step carbonization and CO_2_ activation proceed to form highly porous carbon spheres. These porous carbons are mixed with urea and heated in air to form highly N-doped porous carbon spheres. After CO_2_ activation and urea modification, the resulting carbon spheres possess a narrow PSD and high N content, which are used as a promising adsorbent. These porous carbon spheres exhibit a high CO_2_ (3.03 mmol g^−1^) and CH_4_ (1.3 mmol g^−1^) adsorption capacity, low N_2_ (0.4 mmol g^−1^) uptake, and excellent selectivity for CO_2_/N_2_ (16.48), CO_2_/CH_4_ (7.49), and CH_4_/N_2_ (3.76) binary mixtures under 25 °C and 1 bar. This study hopes to provide viable insight for both porous carbon preparation and CO_2_/CH_4_/N_2_ separation application.

## 2. Experimental Sections

### 2.1. Materials

D-glucose (C_6_H_12_O_6_), cetyltrimethyl ammonium bromide (CTAB), and urea (NH_2_CONH_2_) were purchased from Sinopharm Chemical Reagent Co., Ltd. (Beijing, China). All chemicals were of analytical grade and were used as received without further purification.

### 2.2. Preparation of Glucose-Based Hydrochar Spheres

Glucose-based hydrochar spheres were prepared according to a previously reported method [50]. Briefly, 3.5 g glucose and 0.5 g CTAB were dissolved in 70 mL deionized (DI) water, and stirred with a magnetic stirrer for 2 h at room temperature. Then, the solution was transferred into a Teflon-lined stainless steel autoclave (100 mL), sealed, and maintained at 180 °C for 6 h. Then, the solution was cooled to room temperature. The dark brown precipitate was collected by centrifugation and washed with DI water and pure ethanol several times, and then dried in an oven at 80 °C overnight. Finally, the glucose-based hydrochar spheres were denoted as HSs.

### 2.3. Preparation of Porous Carbon Spheres

The HSs were heated in a tube furnace under Ar atmosphere at a heating rate of 3 °C min^−1^ first to 500 °C with a retention of 3 h, and then to 800 °C. When 800 °C was reached, flowing CO_2_ was introduced to activate the sample for 2 h. Finally, the activated sample was cooled naturally to room temperature in an Ar flow. The obtained activated porous carbon spheres were recorded as ACSs. The ACSs were mixed with urea at a weight ratio of 1:1, heated in air at 350 °C for 2 h, followed by washing with hot water to remove the unreacted urea and dried overnight at 80 °C. The urea-treated ACSs were named ACSs-N.

For comparison, the HSs were first modified by urea, and then carbonized only in Ar; the experimental conditions were the same as those depicted above. The as-obtained carbon spherical products were named NCSs.

### 2.4. Characterizations

Scanning electron microscopy (SEM) was performed on a Hitachi S-4800 instrument (Hitachi, Tokyo, Japan). Transmission electron microscopy (TEM) and high-resolution TEM (HR-TEM) were performed with a JEM-2100 microscope ((JEOL, Tokyo, Japan). Powder X-ray diffraction (XRD) patterns were recorded on a Bruker D8 advanced X-ray diffractometer (Bruker, Madison, WI, USA) using Cu-Kα radiation (λ = 0.15406 nm). Raman spectra were collected using a Renishaw inVia spectrometer (Renishaw, London, UK) with an excitation wavelength of 514 nm. Fourier-transform infrared (FT-IR) spectra were recorded on a Nicolet 5700 (Nicolet, Madison, WI, USA), using the KBr pellet method. Elemental analysis (C, H, and N) was performed on a dry basis using a VarioEL III Elemental Analyzer (Elementar, Hanau, Germany). The surface composition of samples was characterized by X-ray photoelectron spectroscopy (XPS), using a Thermo ESCALAB250Xi (Thermo Fisher Scientific, Waltham, MA, USA). Textural properties of the samples were measured by N_2_ adsorption−desorption isotherms at −196 °C using a Micromeritics ASAP 2420 surface area and porosimetry analyzer (Micromeritics, Norcross, GA, USA). The Brunauer−Emmett−Teller (BET) surface area (*S_BET_*) was calculated using the N_2_ adsorption isotherm data between 0.005 and 0.05 relative pressure, the total pore volume (*V_tot_*) was determined from the amount of N_2_ adsorbed at a relative pressure of ~0.99, and the PSD was calculated using nonlocal density functional theory (NLDFT) from the N_2_ adsorption isotherm. The micropore volume (*V_micro_*, pore widths below 2 nm) and narrow micropore volume (V_1_, pore widths below 1 nm) were calculated by the cumulative pore volume method.

### 2.5. Gas Adsorption Measurements

The adsorption−desorption isotherms for CO_2_, CH_4_, and N_2_ were measured on an intelligent gravimetric analyzer (IGA-002, Hiden, Manchester, UK) at different temperatures of 0, 25, and 45 °C and pressures up to 1 bar. The purity of the used CO_2_, CH_4_, and N_2_ were all 99.999%. Before each adsorption measurement, samples were degassed under 10^−6^ bar at 300 °C for 3 h.

### 2.6. Langmuir−Freundlich (LF) Isotherm Calculation for ACSs-N

The LF model was applied to the CO_2_, CH_4_, and N_2_ adsorption isotherms of the ACSs-N. The LF isotherm equation can be expressed as follows:

(1) $$q=q_{s}\times \frac{b\times p^{n}}{1+b\times p^{n}}$$

where *q* is the amount of gas adsorbed in equilibrium (mmol g^−1^), *p* is the equilibrium pressure (bar), *q_s_* is the saturation capacity (mmol g^−1^), *b* is the affinity constant, or called the LF isotherm constant (bar^−1^), and *n* is a dimensionless parameter reflecting the heterogeneity of adsorbent surfaces.

### 2.7. Calculation of the Selectivity

To investigate the adsorption selectivity of CO_2_/N_2_, CO_2_/CH_4_, and CH_4_/N_2_ on ACSs-N, the selectivity is defined as follows:

*S*_1/2_ = [*x*_1_ /*x*_2_]/[*y*_1_ /*y*_2_], (2)

where *S*_1/2_ is the selectivity factor, and *x*_1_ and *x*_2_ are the absolute adsorbed loadings at a partial pressure of *y*_1_ and *y*_2_, respectively. Ideal adsorbed solution theory (IAST) was used to calculate the binary mixture selectivity via the adsorption isotherms of a single component.

### 2.8. Calculation of the Isosteric Heat of Adsorption (Qst)

The *Q_st_* of CO_2_, CH_4_, and N_2_ was calculated using the adsorption isotherms measured at 0, 25, and 45 °C based on the Clausius−Clapeyron equation:

(3) $$Q_{st}=-RT^{2}{\left(\frac{\partial lnP}{\partial T}\right)}_{q},$$

where *Q_st_* (kJ mol^−1^) is the isosteric heat of adsorption, *P* (kPa) is the pressure, *T* (K) is the temperature, *R* is the gas constant, and *q* (mmol g^−1^) is the adsorbed amount. Integrating Equation (3), with q as a constant, can give

(4) $$lnP=\frac{Q_{st}}{RT}+C,$$

where *C* is an integral constant. The *Q_st_* of CO_2_, CH_4_, and N_2_ were calculated via the slopes of the linear plots of ln*P* vs. *1*/*T* by using the CO_2_, CH_4_, and N_2_ equilibrium isotherms data.

## 3. Results and Discussion

**Figure 2:**
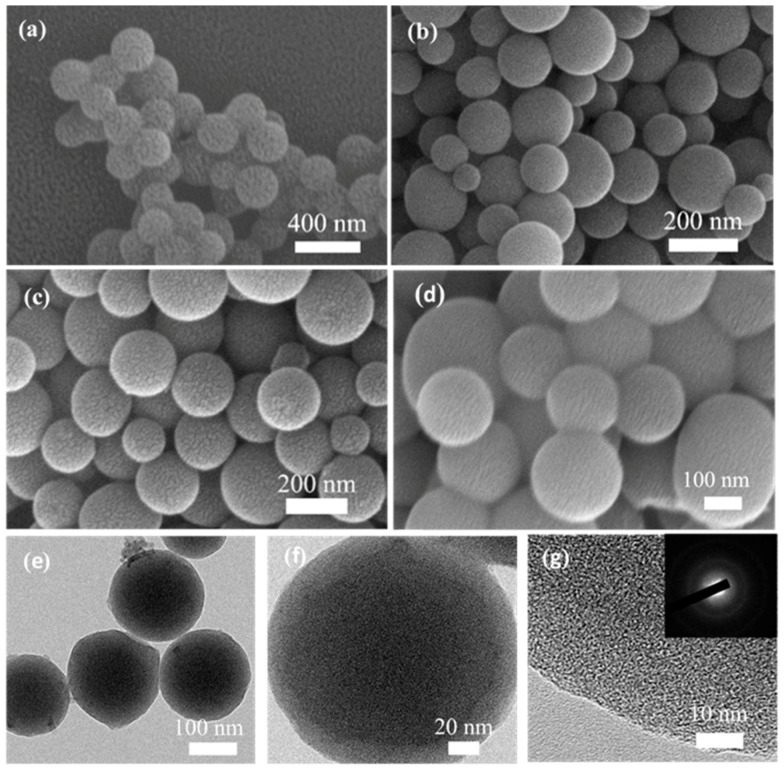
SEM images of (**a**) HSs, (**b**) ACSs, (**c**) ACSs-N, and (**d**) NCSs. TEM images of (**e**) low magnification, (**f**) high magnification, and (**g**) high-resolution (HR)-TEM for ACSs-N. The inset in Figure 2g is the selected-area electron diffraction (SAED) pattern.

The morphology and structural details of the as-prepared samples were examined by SEM and TEM, as shown in Figure 2. In the hydrothermal process, soluble macromolecules polymers are first formed from the aromatization among the glucose molecules [51,52,53]. When the concentration of these polymers reaches a critical supersaturation point, insoluble nuclei are formed by cross-linking the macromolecules with free glucose monomers [54,55]. Then, with the help of CTAB, these nuclei adsorb surrounding molecules, leading to the growth of uniform hydrothermal carbonaceous microspheres. [50,56]. As can be seen in Figure 2a, the as-obtained hydrochar spheres (HSs) exhibited a regularly spherical shape and rough surface, which had an average diameter of about 360 nm. After CO_2_ activation and urea modification, the spherical shape of the ACSs (Figure 2b) and ACSs-N (Figure 2c) could be maintained well. However, the average diameter of the spheres gradually decreased to 310 nm for ACSs and 180 nm for ACSs-N, which should be attributed to the shrinkage of the carbon skeleton in the high-temperature treatment and urea modification. Additionally, the NCSs showed a spherical morphology with an average size of 200 nm (Figure 2d). It is worthwhile noting that the surface of the ACSs-N was much rougher than that of the HSs, which should be attributed to the CO_2_ activation and urea treatment. This rough surface texture is beneficial for the gas diffusion from various orientations and further contact with the active sites [1]. TEM examination was further used to confirm the spherical morphology and structure for the ACSs-N (Figure 2e). The TEM image of an individual sphere for ACSs-N shows a dark kernel with obvious interfaces with an outer part that has lower opacity (Figure 2f). This indicates that the CO_2_ activation at high temperature uniformly and radially etched from the outside to the inside. Furthermore, the randomly distributed wormhole-like micropores can be obviously seen in the HR-TEM image (Figure 2g), which is the feature of amorphous carbon. The selected-area electron diffraction (SAED) pattern exhibits typical diffuse rings (inset in Figure 2g), also reflecting the amorphous nature of carbon.

**Figure 3:**
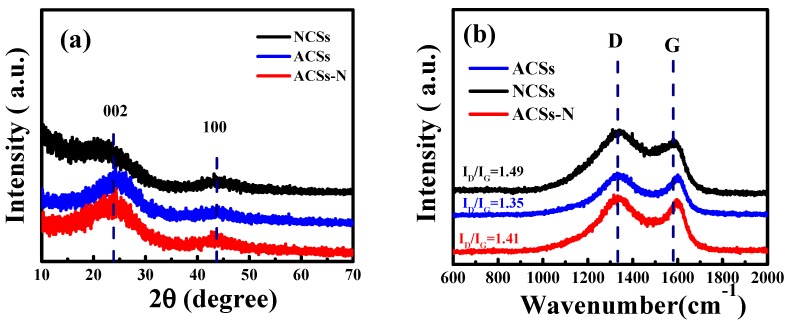
(**a**) XRD patterns and (**b**) Raman spectra of the porous carbons NCSs, ACSs, and ACSs-N.

The phase structure of the as-prepared porous carbons NCSs, ACSs, and ACSs-N was examined by XRD and Raman spectroscopy. As displayed in Figure 3a, two typical broad peaks at around 23° and 43° were found for these samples, which are usually assigned to (002) and (100) diffraction patterns of the amorphous carbon [21,30]. This result is well in accordance with the Raman spectra, as shown in Figure 3b. The Raman spectra show that all these porous carbons exhibited two obvious bands at around 1330 cm^−1^ (D-band) and 1580 cm^−1^ (G-band), with the D-band associated with the disordered carbon structure and the G-band related to the graphitic carbon structure [57,58]. The relative intensity ratio between the D and G band (*I*_D_/*I*_G_) represents the degree of defects in carbon materials, which is determined by the integral area under the peak for the D- and G-bands [21,23]. The *I*_D_/*I*_G_ value for NCSs, ACSs-N, and ACSs was calculated to be 1.49, 1.41, and 1.35, respectively, which suggest that more defects were generated by urea treatment.

**Figure 4:**
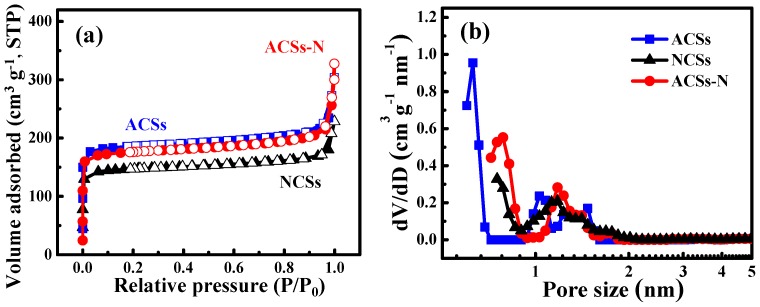
(**a**) N_2_ adsorption−desorption isotherms and (**b**) pore size distribution (PSD) curves of the porous carbons NCSs, ACSs, and ACSs-N.

The texture structure of the as-prepared porous carbons NCSs, ACSs, and ACSs-N was characterized by N_2_ sorption at −196 °C. The N_2_ adsorption−desorption isotherms, PSD curves, and corresponding parameters are illustrated in Figure 4 and Table 1. All the porous carbons exhibited a typical type-I isotherm without a hysteresis loop (Figure 4a) according to the International Union of Pure and Applied Chemistry (IUPAC) classification, which is characteristic for microporous materials [59]. The steep rise in these isotherms at low relative pressure (*P*/*P*_0_ < 0.01) followed by a sharp knee is due to the capillary filling of micropores [21,30]. However, there still exists some difference in the isotherms for these porous carbons prepared by different strategies. The N_2_ uptake amount of these porous carbons is one obvious difference following the order: ACSs > ACSs-N > NCGs, which is in accordance with their *S_BET_*, pore volume, and PSD curve. The corresponding textural parameters are summarized in Table 1, and the ACSs had the largest *S_BET_*, *V_tot_*, *V_micro_*, and *V*_1_ of 748 m^2^ g^−1^, 0.47 cm^3^ g^−1^, 0.27 cm^3^ g^−1^, and 0.21 cm^3^ g^−1^, respectively, while the *S_BET_* of ACSs-N decreased to 697 m^2^ g^−1^, and the *V_tot_*, *V_micro_*, and V_1_ decreased to 0.46 cm^3^ g^−1^, 0.25 cm^3^ g^−1^, and 0.17 cm^3^ g^−1^, respectively, which is attributed to the urea treatment causing some carbon skeleton collapse and micropore coalescence of the carbon sphere interior. However, the NCSs obtained by urea treatment and high-temperature carbonization only possessed an *S_BET_*, *V_tot_*, *V_micro_*, and *V*_1_ of 581 m^2^ g^−1^, 0.35 cm^3^ g^−1^, 0.21 cm^3^ g^−1^, and 0.13 cm^3^ g^−1^, respectively, which are much lower than those of ACSs and ACSs-N owing to the absence of CO_2_ activation. The textural difference for these porous carbons is further confirmed by the corresponding PSD curves (Figure 4b). The PSD curve for ACSs displays one intensity peak at 0.63 nm and two weak peaks at 1.0 and 1.5 nm, respectively. For the ACSs-N, one relatively strong peak is concentrated at 0.78 nm, while the other relatively weak peak is concentrated at 1.17 nm. Compared to ACSs, the broadened pore size and weakened peak intensity for ACSs-N are due to the skeleton collapse and micropore coalescence of the carbon sphere interior caused by the urea treatment. As for NCSs, without CO_2_ activation, the PSD curve exhibits inferior strength peaks around 0.75 and 1.18 nm, respectively. The theoretical and experimental results have shown that the narrow PSD will not only provide accommodation space for gas molecules but also confine them in micropores though the Van der Waals’ force [23,37]. In addition, the narrow PSD will give a promising merit for separating gas molecules that are similar in size, such as CO_2_, CH_4_, and N_2_ (kinetic diameters are 3.3, 3.8, and 3.64 Å, respectively) [22,38]. Though the microporous structure of ACSs-N is not the best in these porous carbon spheres, it is still suitable for gas capture and separation.

**Figure 5:**
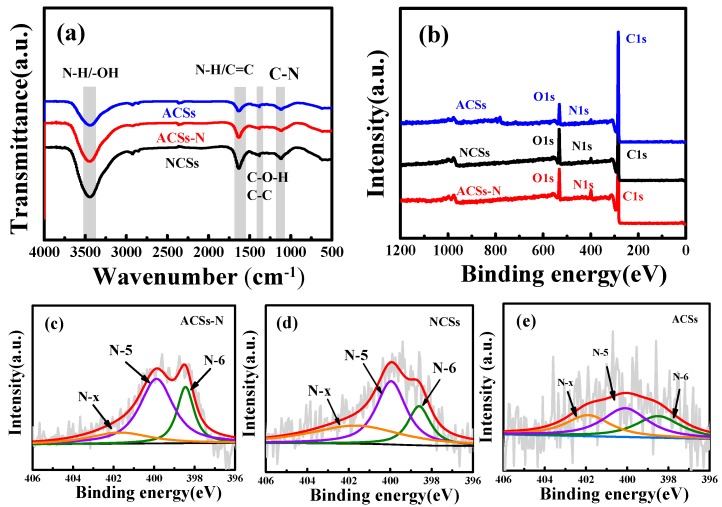
(**a**) FT-IR spectra and (**b**) X-ray photoelectron spectroscopy (XPS) survey spectra of the porous carbons ACSs, NCSs, and ACSs-N. The N1s high-resolution spectra of (**c**) ACSs-N, (**d**) NCSs, and (**e**) ACSs.

The chemical compositions of the as-prepared porous carbons are listed in Table 1. According to the element analysis, ACSs had a negligibly low *N* content of 1.10 wt%, which was just from the CTAB. After urea treatment, the ACSs-N possessed a much higher *N* content of 6.50 wt.%. This result indicates that *N* could be integrated into the carbon skeleton by urea treatment, while the NCSs had a very high *N* content of 11.48 wt.%, indicating that much more *N* could be integrated into the hydrochar via urea treatment. Even under high-temperature carbonization, a mass of *N* could also be reserved in the carbon skeleton. Although, the N content of ACSs-N was not as high as that of NCSs, which is still desirable among the *N*-doped porous carbons compared to those previously reported [2,21,23,58,60]. The FT-IR was used to characterize the bonding configuration of the *N* atom in the porous carbons, as shown in Figure 5a. The adsorption bands at around 3430 cm^−1^ are ascribed to the *N*−*H* and/or −OH stretching vibration [21,30,31]. The band at about 1620 cm^−1^ can be attributed to *N*–*H* in-plane deformation vibrations or *C*=*C* stretching vibrations [21,31], while the band at around 1110 cm^−1^ corresponds to the *C*–*N* stretching vibrations [23,31]. It is noteworthy that the strength of the N-related bands for these porous carbons is in the order of NCSs>ACSs-N>ACSs, which is in accordance with the element analysis result. Additionally, the *C*−*O*−*H* stretching and *C*−*C* vibration can be observed at 1384 and 1352 cm^−1^, respectively [21]. The adsorption bands of the FT-IR spectrum also revealed that the chemistry components of *C*, *N*, *H*, and *O* were in the samples, which is accordance with the element analysis. The compositions of the as-produced porous carbon samples were further determined by XPS. The XPS survey spectra in Figure 5b corroborate the existence of *C*, *N*, and *O* in the samples. Considering the importance of the heteroatom *N*, the nature of the *N* species on the carbon surface of ACSs-N, NCSs, and ACSs was investigated. As shown in Figure 5c–e, three sub-peaks are visible in the deconvoluted XPS N1s spectra: Pyridinic-*N* (*N*-6) at 398.5 eV, pyrrolic-/pyridonic-*N* (*N*-5) at 399.9 eV, and pyridine-*N*-oxide (*N*-*X*) at 402 eV, respectively [21,30,39,58]. The presence of *N* can not only provide Lewis basic active sites, but also increase the carbon framework polarity, which is beneficial for CO_2_/CH_4_/N_2_ selectivity adsorption [21,23,35,37]. It should be stressed that *N*-5 was the main type of *N* species in the carbons, which is beneficial to boosting the CO_2_ capture [21,35,60]. Moreover, the high-resolution spectra of C1s (Figure S1a–c) can be deconvoluted into five peaks, which are respectively attributed to *C*−*C* (284.5 eV), *C*−*O* (285.4 eV), *C*−*N* (285.9 eV), *C*=*O* (287.5 eV), and *O*=*C*−*O* (289.4 eV) [61,62,63,64]. The split spectra of O1s (Figure S1d–f) located at 532.5 and 533.4 eV can be assigned to *C*=*O* and *C*−*O*, respectively [63,65].

**Figure 6:**
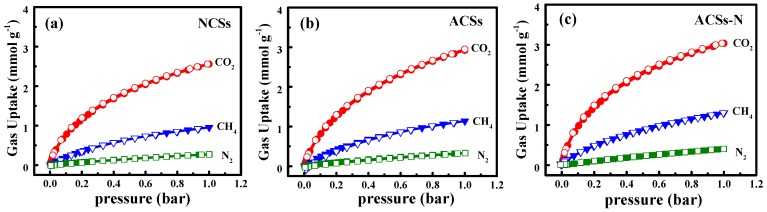
Adsorption (solid) and desorption (open) isotherms of CO_2_, CH_4_, and N_2_ on (**a**) NCSs, (**b**) ACSs, and (**c**) ACSs-N at 25 °C.

The single-component adsorption−desorption isotherms of CO_2_, CH_4_, and N_2_ on NCSs, ACSs, and ACSs-N at 25 °C and up to 1 bar are given in Figure 6. All the adsorption and desorption isotherms completely overlap with each other without any hysteresis, suggesting that the adsorbed molecules can be fully removed during the desorption process. Thus, the adsorption process is considerably reversible and these porous carbon adsorbents can be easily regenerated under vacuum without any heat energy input. Based on the adsorption isotherms, it is clear that all these porous carbons exhibited preferential adsorption of CO_2_ over CH_4_ and N_2_, which should be ascribed to the higher quadrupole moment and polarizability of CO_2_ molecules than those of CH_4_ and N_2_ [21,66]. CH_4_ is much more favorably adsorbed than N_2_, because of its higher polarizability than that of N_2_ molecules [6,66]. Although, the N_2_ molecule exhibits a higher quadrupole moment than that of CH_4_, which is of less influence than the difference in polarizability [10]. Moreover, the isotherms for CO_2_ and CH_4_ modestly curved, and neither reached their saturated adsorption capacity over the entire pressure range examined here. This means that much more CO_2_ and CH_4_ could be adsorbed on the adsorbents at high pressure, while the isotherms for N_2_ were almost linear, meaning a weak interaction between N_2_ and these carbon adsorbents.

The gas capacities of the NCSs, ASCs, and ACSs-N at 25 °C and 1 bar are summarized in Table 2. Among them, ACSs-N displayed the best performance, and its CO_2_ capacity was 3.03 mmol g^−1^ at 25 °C and 1 bar, which is comparable to or higher than those of other state-of-the-art porous carbon adsorbents (see Table S1). It is well known that the favorable CO_2_ capacity arose from two critical factors: (i) Highly microporous structure, especially the narrow micropores (<1 nm), which could greatly accommodate CO_2_ molecules into pores; and (ii) heteroatom incorporation, especially N-doping, which could increase the surface basicity to enhance the bonding force with acidic CO_2_ molecules [23,28,29,32]. However, the high micro-porosity is often inverse with the *N* content. The NCSs had the highest *N* content, but the microporous structure was poor. As for the ACSs-N, the microporous structure was excellent, while the *N* content was very low. Thus, in the same condition, the CO_2_ capacity for NCSs and ACSs was just 2.55 and 2.92 mmol g^−1^, respectively, and both were inferior to that of ACSs-N. The high CO_2_ capacity for ACSs-N should be attributed to the synergistic effect between suitable microporous structure, especially the narrow micropores (<1 nm), and desirable *N* content. Additionally, special attention should be paid to the CH_4_ capacity owing to its importance for new energy applications. The CH_4_ uptakes of the three porous carbon samples under ambient conditions (25 °C and 1 bar) follow the order of ACSs-N (1.30 mmol g^−1^) > ACSs (1.14 mmol g^−1^) > NCSs (0.95 mmol g^−1^). ACSs-N exhibited the best CH_4_ uptake performance among these porous carbon spheres, which is comparable to or higher than the values in reported in the literature for porous adsorbents (see Table S1). The N_2_ uptakes of the carbon samples follow the same order as those of CO_2_ and CH_4_: ACSs-N (0.4 mmol g^−1^) > ACSs (0.33 mmol g^−1^) > NCSs (0.27 mmol g^−1^). The high CO_2_ and CH_4_ adsorption capabilities of ACSs-N motivate us to further investigate their adsorptive separation performance for CO_2_/CH_4_/N_2_.

**Figure 7:**
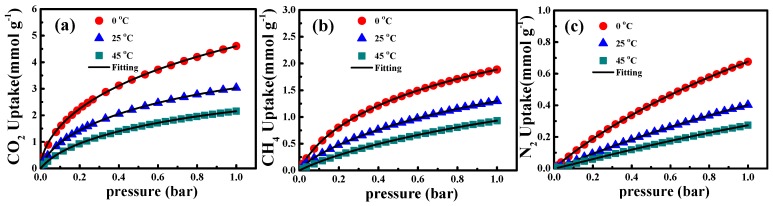
Adsorption isotherms of (**a**) CO_2_, (**b**) CH_4_, and (**c**) N_2_ on ACSs-N. The marker points represent the experimental data, while the black solid lines correspond to Langmuir−Freundlich equation fittings.

The pure gas adsorption isotherms of CO_2_, CH_4_, and N_2_ on ACSs-N at three temperatures (0, 25, and 45 °C) are plotted in Figure 7a–c, respectively. From these figures, it can be seen that temperature and pressure had opposite effects on the gas (CO_2_, CH_4_, and N_2_) adsorption capacity. Indeed, the gas adsorption capacity increased with the pressure, which is in accordance with the fact that pressure is the thermodynamic driving force of the adsorption process [25]. On the contrary, the amount of adsorbed gas was reduced with the increase in adsorption temperature, which is in agreement with the adsorption process being exothermic [25]. The adsorption mechanisms for these three gases (CO_2_, CH_4_, and N_2_) were different. The critical temperatures of CH_4_ and N_2_ were 126 and 124 K, respectively, which are lower than the experimental temperature. Therefore, the adsorption of CH_4_ and N_2_ was monolayer supercritical adsorption. However, the critical temperature of CO_2_ was equal to 304.3 K, which is closer to the experimental range. Therefore, the adsorption process included supercritical adsorption and subcritical adsorption. The Langmuir−Freundlich (LF) model is a combination of the Langmuir and the Freundlich isotherm models for predicting the behavior of heterogeneous adsorption systems [25]. As the N-doped porous carbons obtained in this work were not homogeneous, all the isotherms correlated with the LF model. The coefficient of determination R^2^ and the parameters are listed in Table 3. All R^2^ values in Table 3 are above 0.999, indicating that the experimental data can agree well with the LF equation. It is also clear from Figure 7 that there is good agreement between the model fitting and the experimental adsorption data. To highlight the gas adsorption performance of ACSs-N, we further measured the gas adsorption isotherms of CO_2_, CH_4_, and N_2_ on ACSs-N under high pressure (Figure S2). The gas capacities of ACSs-N at different temperatures (0, 25, and 45 °C) at 19 bar are summarized in Table S2. From Figure S2, it can be seen that the gas uptakes increased very slow when the pressure reached 16 bar, the adsorption isotherms almost reaching plateau. The high-pressure adsorption data agree well with the LF modeled fittings, further indicating the correct LF equation parameters. 

**Figure 8:**
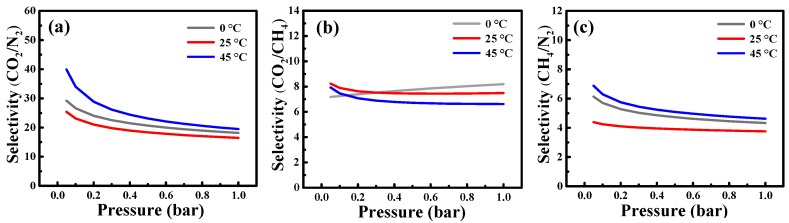
IAST-predicted adsorption selectivities of binary mixtures for (**a**) CO_2_/N_2_ (15/85), (**b**) CO_2_/CH_4_ (10/90), and (**c**) CH_4_/N_2_ (30/70) on ACSs-N.

The ideal adsorbed solution theory (IAST) proposed by Myers and Praunitz [67] is a thermodynamic approach assuming the adsorbed phase forms an ideal solution, where there are no interactions between the adsorbate molecules, and the spreading pressures of the components are equal at constant temperature [68]. IAST has been widely used to examine the binary gas mixture selective adsorption behavior from pure component isotherms [69,70,71,72]. Herein, the LF model was combined with IAST to predict the selectivity of ACSs-N for binary mixtures (CO_2_/N_2_, CO_2_/CH_4_, and CH_4_/N_2_). The gas pairs and proportions investigated in this work, CO_2_/N_2_ (15/85), CO_2_/CH_4_ (10/90), and CH_4_/N_2_ (30/70), were designed to typical flue gas, and energy-related mixed gas (such as CBM, natural gas, and biogas). The selectivity for each binary mixture at 0, 25, and 45 °C on ACSs-N is plotted as a function of total bulk pressure in Figure 8. For a binary mixture of CO_2_ and N_2_, the selectivity decreased with the pressure, obtaining about 18.18 (0 °C), 16.48 (25 °C), and 19.49 (45 °C) at 1 bar (Figure 8a). The CO_2_/N_2_ selectivity displayed by ACSs-N was higher those reported on sOMC (12.7 at 0 °C and 11.3 at 25 °C) [6], WNPC-3 (16 at 25 °C) [40], AC-PAIN-F (18.97 at 25 °C), and AC-PANI-S (6.1 at 25 °C) [73], in the similar condition. Figure 8b shows that the CO_2_/CH_4_ selectivity slightly increased with the pressure increase at 0 °C, whereas it decreased with the pressure increase at 25 and 45 °C. Under 1 bar, CO_2_/CH_4_ selectivities of 8.19, 7.49, and 6.62 were reached on ACSs-N at 0, 25, and 45 °C, respectively. They surpassed the values reported for sOMC (3.4 and 2.9 at 0 and 25 °C, respectively) [6] and SNMC-2-600 (6.3 and 4.3 at 0 and 25 °C, respectively) [23]. When it comes to the CH_4_/N_2_ separation, the selectivity of CH_4_ over N_2_ gradually decreased as the pressure increased, similar to the case of CO_2_/N_2_ selectivity, as shown in Figure 8c. At 1 bar, the CH_4_/N_2_ selectivity obtained at 4.32 (0 °C), 3.76 (25 °C), and 4.62 (45 °C) was larger or comparable to those found on many porous carbons including sOMC (3.8 at 25 °C) [6], OTSS-2-450 (4.9 at 25 °C) [21], SNMC-2-600 (4.6 and 4.2 at 0 and 25 °C) [23], and ClCTF-1-400 (4.6 at 25 °C) [37]. These comparison results suggest the great potential of ACSs-N in CO_2_/CH_4_/N_2_ adsorptive separation systems.

Isosteric heat of adsorption (Qst) is an important thermodynamic parameter to evaluate the interaction between adsorbate gas molecules and an adsorbent [5,6,38], which can be estimated from the adsorption isotherms at different temperatures by using the Clausius−Clapeyron equation.

**Figure 9:**
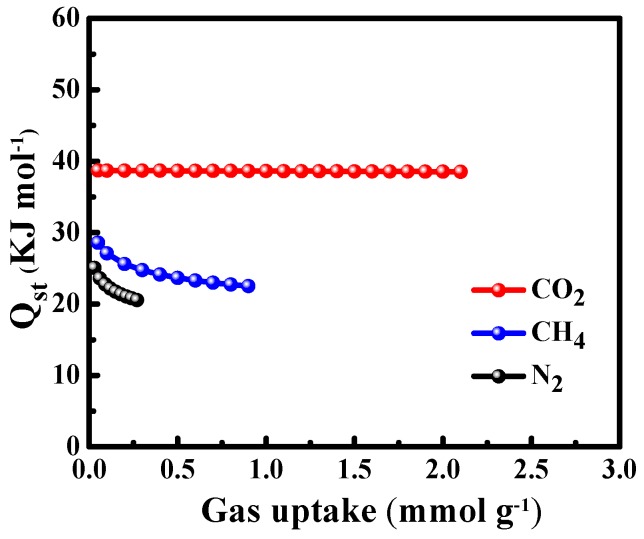
Isosteric heats of adsorption for CO_2_, CH_4_, and N_2_ on the ACSs-N.

Figure 9 shows the Qst of CO_2_, CH_4_, and N_2_ on ACSs-N. It can be seen that the Qst of CH_4_ and N_2_ gradually decreased with the increase in surface coverage; however, the Qst of CO_2_ was almost unchanged. A decrease in Qst with gas loading is characteristic of heterogeneous adsorbents; whereas a constant Qst with gas loading indicates a balance between the strength of cooperative gas−gas interactions and the degree of heterogeneity of gas−solid interactions [74,75,76]. The ACSs-N had a heterogenous surface for the adsorption of CO_2_, CH_4_, and N_2_ with the Qst range of 38.7–38.5 (CO_2_), 28.6–22.5 (CH_4_), and 25.0–20.6 kJ mmol^−1^(N_2_), respectively, from low coverage to saturation. The Qst follows the order of CO_2_>CH_4_>N_2_, suggesting that the interaction of CO_2_ with ACSs-N was stronger than that of CH_4_ with N_2_; meanwhile, the interaction of CH_4_ with ACSs-N was stronger than that of N_2_. Notably, the highest Qst for CO_2_ was still in the value range of the physisorption process, which means that the desorption process was simple and reversible. The above Qst characteristics make ACSs-N a promising adsorbent for CO_2_/CH_4_/N_2_ separation.

## 4. Conclusions

In summary, the ACSs-N were prepared by CO_2_ physical activation of glucose-derived hydrochar and urea treatment, which was used for efficient CO_2_ and CH_4_ adsorption and CO_2_/CH_4_/N_2_ adsorptive separation. The obtained ACSs-N possessed a large *S_BET_* of 697 m^2^ g^−1^, suitable *V_tot_*, *V_mico_*, and *V*_1_ of 0.46, 0.25, and 0.21 m^3^ g^−1^, respectively, and desirable *N* content of 6.5 wt.%. On account of the synergistic effect between suitable microporous structure and desirable *N* content, under 25 °C and 1 bar, the capacities of ACSs-N for CO_2_ and CH_4_ were 3.03 and 1.3 mmol g^−1^, respectively, and the IAST predicted selectivities for CO_2_/N_2_ (15/85), CO_2_/CH_4_ (10/90), and CH_4_/N_2_ (30/70) binary mixtures reached 16.48, 7.49, and 3.76, respectively. These results make ACSs-N a highly promising adsorbent for CO_2_ and CH_4_ capture and CO_2_/CH_4_/N_2_ adsorptive separation. Given the sustainability and environmental friendliness of the raw material, this work provides a useful approach to prepare relatively inexpensive *N*-doped porous carbon adsorbents for industrial applications.

## Figures and Tables

**Table 1:** Textural parameters and chemical compositions of the porous carbons.

Sample	Textural Parameters				Chemical Compositions			
	*S_BET_*^a^ (m^2^ g^−1^)	*V_total_*^b^ (cm^3^ g^−1^)	*V_micro_*^c^ (cm^3^ g^−1^)	*V*_1_^d^ (cm^3^ g^−1^)	*C*^e^ (wt %)	*N*^e^ (wt%)	*H*^e^ (wt %)	*O*^f^ (wt%)
ACSs	748	0.47	0.27	0.21	83.84	1.10	0.04	15.02
ACSs-N	697	0.46	0.25	0.17	75.15	6.50	0.05	18.30
NCSs	581	0.35	0.21	0.13	67.78	11.48	1.24	19.50

^a^ Specific surface area calculated by Brunauer−Emmett−Teller (BET) method; ^b^ total pore volume obtained at P/P_0_ ~ 0.99; ^c^ cumulative pore volume calculated in the range of pore widths up to 2 nm; ^d^ cumulative pore volume calculated in the range of pore widths up to 1 nm; ^e^ obtained from *C*, *H*, and *N* elemental analysis; ^f^ calculated by difference.

**Table 2:** Summary of the gas capacities of the NCSs, ASCs, and ACSs-N at 25 °C and 1 bar.

Sample	CO_2_ Uptake (mmol g^−1^)	CH_4_ Uptake (mmol g^−1^)	N_2_ Uptake (mmol g^−1^)
NCSs	2.55	0.95	0.27
ACSs	2.92	1.14	0.33
ACSs-N	3.03	1.30	0.40

**Table 3:** Equation parameters for the Langmuir−Freundlich isotherm model on ACSs-N.

Adsorbate	Temp. (°C)	*q*_s_	*b*	*n*	*R* ^2^
CO_2_	273	6.28199	1.84997	0.72764	0.99990
	298	5.97195	1.00124	0.70470	0.99900
	318	5.74151	0.63787	0.72580	0.99900
CH_4_	273	4.53961	0.69512	0.74049	0.99900
	298	4.22490	0.42287	0.78157	0.99990
	318	3.96414	0.29156	0.82416	0.99999
N_2_	273	3.53500	0.24440	0.83413	0.99990
	298	3.18912	0.17080	0.82522	0.99900
	318	2.81950	0.10433	0.94821	0.99990

## Supplementary Materials

The following are available online at https://www.mdpi.com/2079-4991/10/1/174/s1, Figure S1: XPS high-resolution of (a,b,c) C1s and (d,e,f) O1s for the porous carbon samples ACSs-N, NCSs and ACSs, Figure S2: Adsorption isotherms of (a) CO_2_, (b) CH_4_, and (c) N_2_ on ACSs-N at high pressure. The marker points represent the experimental data, while the black solid lines correspond to Langmuir-Freundlich equation fittings, Table S1: The gas adsorption performance for porous materials from reported results, Table S2: Summary of the gas capacities of the ACSs-N under high pressure.
