# Supplementary material for: Sustainable Biomass Glucose-Derived Porous Carbon Spheres with High Nitrogen Doping: As a Promising Adsorbent for CO2/CH4/N2 Adsorptive Separation
Source: Nanomaterials (Basel). 2020 Jan 19;10(1):174. doi: 10.3390/nano10010174 (PMC7023444; doi:10.3390/nano10010174)
Supplement: Supplementary file 1 [file nanomaterials-10-00174-s001.pdf]

Supplementary Information

Sustainable Biomass Glucose-Derived Porous Carbon Spheres with High Nitrogen Doping: As a Promising Adsorbent for CO_2/CH_4/N_2 Adsorptive Separation

Yao Li [1,2,†], Shiying Wang [1,†], Binbin Wang [3,*], Yan Wang [1] and Jianping Wei [1,2,*]

[1] School of Safety Science and Engineering, Henan Polytechnic University, Jiaozuo 454000, China; leayao35@hpu.edu.cn (Y.L.); 211701010023@home.hpu.edu.cn (S.W.); yanwang@hpu.edu.cn (Y.W.)
[2] State Key Laboratory Cultivation Base for Gas Geology and Gas Control, Henan Polytechnic University, Jiaozuo 454000, China
[3] School of Materials Science and Engineering, Henan Polytechnic University, Jiaozuo 454000, China
* Correspondence: wangbb@hpu.edu.cn (B.W.); weijianping@hpu.edu.cn (J.W.);
Tel.: +86-391-398-6901 (B.W.); +86-391-398-7885 (J.W.)

Table S1. The gas adsorption performance for porous materials from reported results.

samples	CO_2 uptake (mmol g^{-1})	CH_4 uptake(mmol g^{-1})	Ref.
	25 °C	25 °C	
**ACSs-N**	**3.03**	**0.93**	**This work**
NPCs-2-500	2.5	----	S1
WNPC-3	2.78	----	S2
AC-PAIN-F	2.69	----	S3
STC-2.5	1.3	----	S4
500-2	3.5	----	S5
SNMC-2-600	4.24	1.57	S6
OTSS-1-550	3.1	0.5	S7
sOMC	2.0	0.9	S8
Ni formate	----	0.82	S9
Cu(hfipbb)(H_2hfipbb)0.5	0.86	0.47	S10
Cu(Me-4py-trz-ia)	----	1.12	S11
MOF-177	----	0.56	S12
MOF-5	----	0.13	S12

Table S2. Summary of the gas capacities of the ACSs-N under high pressure.

**Sample**	**CO_2 uptake (mmol g^{-1})**	**CH_4 uptake (mmol g^{-1})**	**N_2 uptake (mmol g^{-1})**
0 °C	5.87	3.86	2.60
25 °C	5.25	3.40	2.12
45 °C	4.82	3.03	1.78

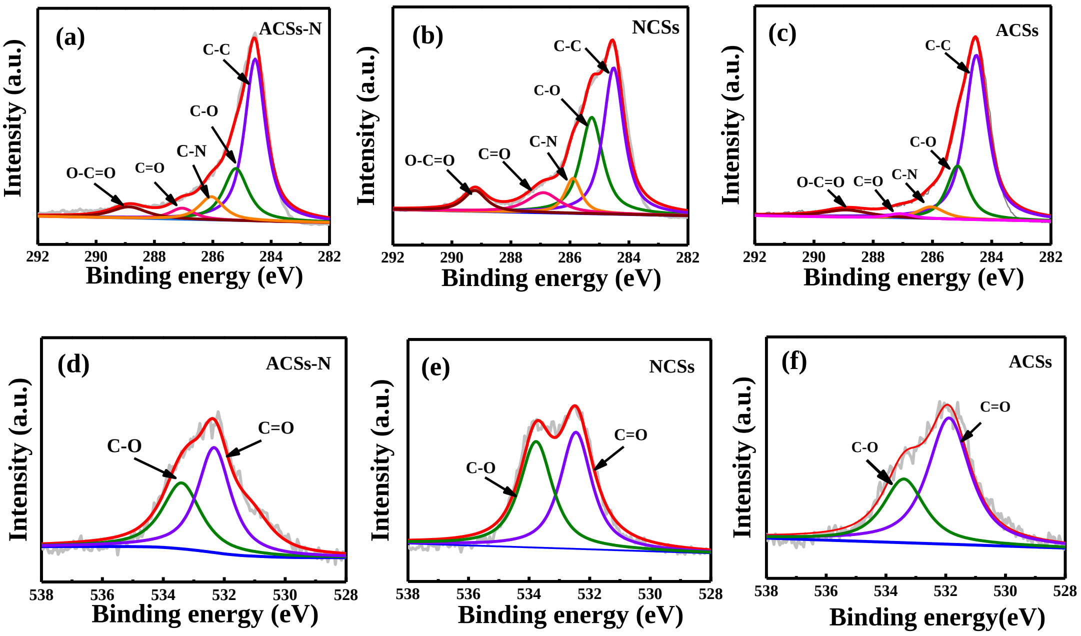

Figure S1. XPS high-resolution of (a,b,c) C1s and (d,e,f) O1s for the porous carbon samples ACSs-N, NCSs and ACSs.

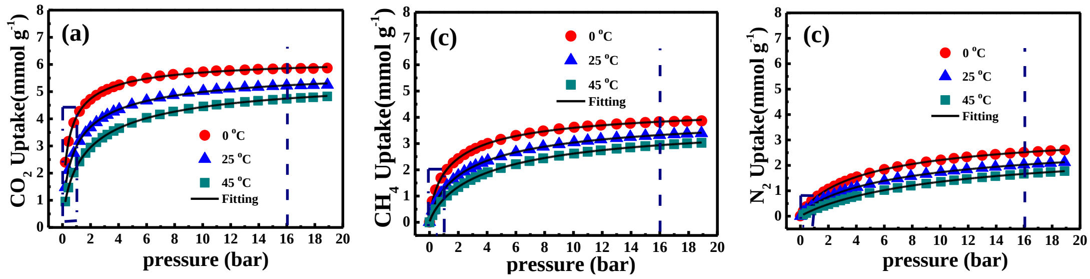

Figure S2. Adsorption isotherms of (a) CO2, (b) CH4, and (c) N2 on ACSs-N at high pressure. The marker points represent the experimental data, while the black solid lines correspond to Langmuir-Freundlich equation fittings.

[1] Wei, H.M.; Qian, W.; Fu, N.; Chen, H.J.; Liu, J.B.; Jiang, X.Z.; Lan, G.X.; Lin, H.L.; Han, S. Facile synthesis of nitrogen-doped porous carbons for CO_2 capture and supercapacitors. *J. Mater. Sci.* **2017**, *52*, 10308-10320.

[2] Li, Y.; Xu, R.; Wang, X.; Wang, B.B.; Cao, J.L.; Yang, J.; Wei, J.P. Waste wool derived nitrogen-doped hierarchical porous carbon for selective CO_2 capture. *RSC Adv.* **2018**, *8*, 19818-19826.

[3] Khalili, S.; Khoshandam, B.; Jahanshahi, M. Synthesis of activated carbon/polyaniline nanocomposites for enhanced CO_2 adsorption. *RSC Adv.* **2016**, *6*, 35692-35704.

[4] Sivadas, D.L.; Narasimman, R.; Rajeev, R.; Prabhakaran, K.; Ninan, K.N. Solvothermal synthesis of microporous superhydrophobic carbon with tunable morphology from natural cotton for carbon dioxide and organic solvent removal applications. *J. Mater. Chem. A* **2015**, *3*, 16213-16221.

[5] Lee, D,Y.; Zhang, C.Y.; Wei, C.; Ashfeld, B.L.; Gao, H.F. Hierarchically porous materials via assembly of nitrogen-rich polymer nanoparticles for efficient and selective CO_2 capture. *J. Mater. Chem. A* **2013**, *1*, 14862.

[6] Zhang, P.X.; Zhong, Y.; Ding, J.; Wang, J.; Xu, M.; Deng, Q.; Zeng, Z.L.; Deng, S.G. A new choice of polymer precursor for solvent-free method: Preparation of N-enriched porous carbons for highly selective CO_2 capture. *Chem. Eng. J.* **2019**, *355*, 963-973.

[7] Zhang, Y.; Liu, L.; Zhang, P.X.; Wang, J.; Xu, M.; Deng, Q.; Zeng, Z.L.; Deng, S.G. Ultra-high surface area and nitrogen-rich porous carbons prepared by a low-temperature activation method with superior gas selective adsorption and outstanding supercapacitance performance. *Chem. Eng. J.* **2019**, *355*, 309-319.

[8] Yuan, B.; Wu, X.F.; Chen, Y.X.; Huang, J.H.; Luo, H.M.; Deng, S.G. Adsorption of CO_2, CH_4, and N_2 on ordered mesoporous carbon: approach for greenhouse gases capture and biogas upgrading. *Environ. Sci. Technol.* **2013**, *47*, 5474-5480.

[9] Guo, Y.; Hu, J.L.; Liu, X.W.; Sun, T.J.; Zhao, S.S.; Wang, S.D. Scalable solvent-free preparation of [$Ni_3(HCOO)_6$] frameworks for highly efficient separation of CH_4 from N_2. *Chem. Eng. J.* **2017**, *327*, 564-572.

[10] Wu, X.F.; Yuan, B.; Bao, Z.B.; Deng, S.G. Adsorption of carbon dioxide, methane and nitrogen on an ultramicroporous copper metal–organic framework. *J. Colloid Interface Sci.* **2014**, *430*, 78-84.

[11] Möllmer, J.; Lange, M.; Möller, A.; Patzschke, C.; Stein, K.; Lässig, D.; Lincke, J.; Gläser, R.; Krautscheid, H.; Staudt, R. Pure and mixed gas adsorption of CH_4 and N_2 on the metal-organic framework Basolite® A100 and a novel copper-based 1,2,4-triazolyl isophthalate MOF. *J. Mater. Chem.* **2012**, *22*, 10274.

[12] Saha, D.; Bao, Z.B.; Jia, F.; Deng, S.G. Adsorption of CO_2, CH_4, N_2O, and N_2 on MOF-5, MOF-177, and Zeolite 5A. *Environ. Sci. Technol.* **2010**, *44*, 1820-1826.
